# Perceptions regarding tobacco control strategies amongst female youth in Delhi, India – a qualitative study

**DOI:** 10.3332/ecancer.2021.1303

**Published:** 2021-10-12

**Authors:** Swati Jain, Vikrant Mohanty, Shipra Arora, Shekhar Grover, Mohit Bidhuri, Neha Singh

**Affiliations:** 1 Consultant - Dental Public Health, Mobile Dental Clinic Project, National Health Mission, Maulana Azad Institute of Dental Sciences, Bahadur Shah Zafar Marg, New Delhi 110002, India; 2 MOI/C (Mobile Dental Clinic Project), Prof. & Head, Department of Public Health Dentistry, Maulana Azad Institute of Dental Sciences, Bahadur Shah Zafar Marg, New Delhi 110002, India; 3 Dental Surgeon, Mobile Dental Clinic Project, National Health Mission, Maulana Azad Institute of Dental Sciences, Bahadur Shah Zafar Marg, New Delhi 110002, India

**Keywords:** tobacco, perception, qualitative research, adolescent, India

## Abstract

Tobacco-related cancer is one of the commonest causes of cancer-related mortality in low- and middle-income countries. As per Global Youth Tobacco Survey-4, India; nearly one-fifth of students aged 13–15 used any form of the tobacco product. Tobacco related challenges have been countered through various tobacco control strategies; however, ignorance and non-compliance to tobacco control strategies to combat the tobacco epidemic shield the tobacco industry in India as well. There is limited literature on perception of tobacco use and tobacco control strategies amongst female youth. Hence, the present study aimed to assess tobacco control perception and perceived challenges through a qualitative approach amongst the youth of Delhi, India. Thematic analysis design of qualitative research was used amongst students of the Senior Secondary (Class XII) at a Government School of Delhi. One-to-one in-depth interviews were carried out for 6–7 individual participants in a day depending upon their availability. A summative content analysis was conducted of all the responses obtained by a data coder who was blinded to the identity of the respondent. A total of 82 school children participated in the study. The majority (82.9%, *N* = 68) of the participants felt that ‘Tobacco is dangerous to health’ and 41.5% (*N* = 34) of the participants were aware of some of the existing tobacco control laws in the country. Around 53.7% (*N* = 44) of the study respondents considered existing tobacco related laws to be ineffective. Regarding the perceived challenges; 31.7% (*N* = 26) of the respondents considered the lack of strict tobacco control laws and punishment strategies as the main factors. The findings from this study substantiated the focus on school based tobacco control strategies. The participants were well appraised regarding the impending danger of tobacco use and dynamic involvement of youth in tobacco control policies is the need of the hour.

## Introduction

Tobacco is one of the leading grounds for global health distress [1]. Tobacco-related cancer is one of the commonest causes of cancer-related mortality in low- and middle-income countries. More than 8 million deaths are attributed to tobacco use globally [1, 2].

In India, nearly 267 million people in the age group of 15 years and above are tobacco users. India still contributes to the highest number of tobacco consumers globally [3]. Almost a million deaths in India are attributed to tobacco consumption, still, the tobacco consumption amongst youth and adults is as high as 0.9 million adults (15+ years old) [4]. The World Bank has reported that nearly 82,000–99,000 children and adolescents all over the world begin smoking every day [5]. As per Global Youth Tobacco Survey-4; nearly one-fifth of the students aged 13–15 used any form of tobacco product and use of any form of tobacco was higher amongst boys than girls [6]. National Family Health Survey-4 also highlighted that 1.6% of the females aged 15–19 years, who participated in the survey consumed some form of tobacco either smoked or non-smoked. Tobacco use has been gradually mounting amongst females especially in developing countries. It has led to a rise in tobacco-related morbidity and mortality amongst females [7].

Surveillance of tobacco use amongst female youth in several countries has revealed that the problem is of equal concern in developed and developing countries [8]. Previous studies done by Lovato *et al* [9] have further emphasised that youth is majorly influenced by advertising, promotion and marketing efforts of the tobacco industry than adults.

Tobacco-related challenges have been countered through various tobacco control strategies in various parts of the world including India. Ignorance and non-compliance with tobacco control strategies to combat the tobacco epidemic shield the tobacco industry in India as well ensuring increasing tobacco dependence especially amongst youth. The Government of India enacted in 2004 its comprehensive tobacco control law called Cigarettes and other Tobacco Products Act 2003 (COTPA 2003) (prohibition of advertisement and regulation of trade and commerce, production, supply and distribution), to reduce tobacco use [10, 11]. Furthermore, the Ministry of Health and Family Welfare has issued guidelines for Tobacco-Free educational institutions to ensure a tobacco-free school environment [12].

Preventing tobacco product use amongst the youth is critical for the success of tobacco control legislation and other strategies. However, there is limited literature on the tobacco-related perception on the use and tobacco control strategies, especially amongst female youth. Hence, the present study aimed to assess tobacco control perception and perceived challenges through a qualitative approach amongst the youth of Delhi, India.

## Methods

### Study design

Phenomenology type of qualitative research design was conducted. Thematic analysis of the data was used to understand and analyse the discernible and perceived challenges towards tobacco control strategies amongst students of Senior Secondary (Class XII) of a Government School of Delhi. The study design was following COREQ Checklist [13].

### Participants

The study participants were recruited from a Government Girls’ Senior Secondary school in Delhi, India from students between the ages of 16 and 17. Purposive sampling was used to select the students as recommended by the school teacher.

### Study instrument

A semi-structured interview guide was prepared based on an in-depth literature review to assess current perceptions and challenges towards tobacco control strategies [5, 7–10]. The guide focused on knowledge, tobacco control perception and perceived challenges, and future participation towards tobacco control strategies. The guide was then validated by Qualitative experts at the Maulana Azad Institute of Dental Sciences before the collection of data. Validation of the interview guide was done by using cumulative techniques. Cumulative validation requires cross-referencing of accessible literature to match the findings [14]. Reliability was ascertained by preserving records of interviews of the respondents of the study. The interview guide was then pilot tested on two students randomly selected from the selected school and was modified accordingly.

The interview consisted of three sections. Section 1 focused on their consciousness towards tobacco and its effect on health. The second section dealt with their perceptions regarding tobacco control strategies in India. Section 3 assessed their views towards their role and their active participation in tobacco control strategies in schools. The audio recording was done and field notes were prepared by a recording clerk for all the interviews.

One-to-one in-depth interview was taken for 6–7 individual participants in a day depending upon their availability. The interview was conducted for 15 working days in January 2020. Interviews were conducted in the school premises in a separate room maximum of 30 minutes each by a single investigator using a pilot-tested interview guide. The students were interviewed till response saturation was encountered and a total of 82 (*N* = 82) study participants were included in the study. Participants were allowed to leave the study at any time during the interview session.

### Data analysis

Summative content analysis was conducted considering the responses from the study population and the sensitivity of the topic concerned. The responses were obtained by a data coder who was blinded to the identity of the respondent. The frequency of appearance of each coding was analysed using a frequency count. The coding tree was prepared for individual keywords derived for each question. The responses were then categorised and put into specific themes all exclusive of each other. The methodology employed was under the supervision of the subject expert to ensure its rigour.

The keywords were so selected/derived to highlight the understanding of each section. The keywords derived were reviewed by the subject expert and in consensus with the authors.

To check the consistency of the keywords, repeat coding and matching were done for every fifth respondent by an independent coder. All the keywords were then entered in a Microsoft Excel sheet for respective questions in each section. The frequency trend of the observed keywords was then analysed using SPSS version 21.

## Results

A total of 82 school children of class 12 from the Government Girls School of Delhi participated in the study. The mean age of the study participants was 16.9 ± 1.2 years. Many respondents gave multiple responses to individual questions. However, these answers were clubbed into major responses, as they had similar implications.

### Knowledge regarding tobacco use and tobacco control strategies

A majority (82.9%; *N* = 68) of the participants felt that ‘Tobacco is dangerous to health’ while 4.8% and 2.4% of the participants considered that tobacco affects family and environment (Table 1).

Regarding the awareness related to tobacco control laws; 41.5% (*N* = 34) of the participants were aware of the tobacco control laws. On enquiring about the details of the various sub-sections of the tobacco-related legislation (Table 1B); the majority of the participants, i.e. 34.14% (*N* = 28) of the respondents were aware of COTPA Section 4 regarding the ban of smoking in public places.

### Perceived barriers towards tobacco control strategies in India

**Figure 1.**
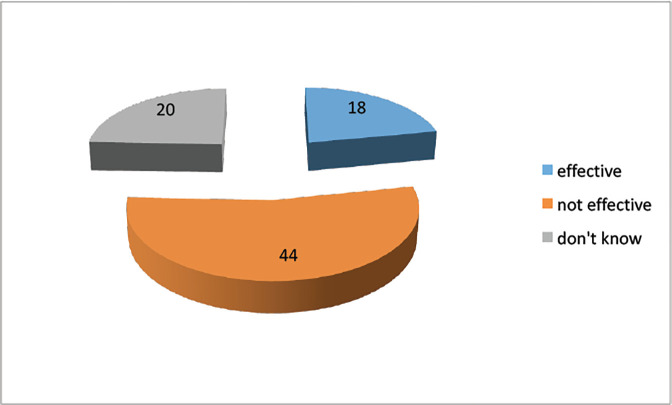
Do you think tobacco control laws are effective in our country?

53.7 % (*N* = 44) of the study respondents considered existent tobacco-related laws to be ineffective (Figure 1).

When enquired in detail regarding the perceived challenges in the implementation of tobacco-related legislations (Table 2); 31.7% (*N* = 26) of the respondents considered the lack of strict tobacco control laws and punishment strategies to be responsible for the complacent attitude amongst the masses.

### Contribution towards tobacco control strategies at the school level

Most of the study respondents (89%; *N* = 73) suggested that various tobacco-related strategies should be dissipated at the school level only. They were further enquired the reason behind this (Table 3); wherein 43.9% (*n* = 36) considered it optimum to increase awareness and bring positive behaviour change during the early phase of life.

**Figure 2.**
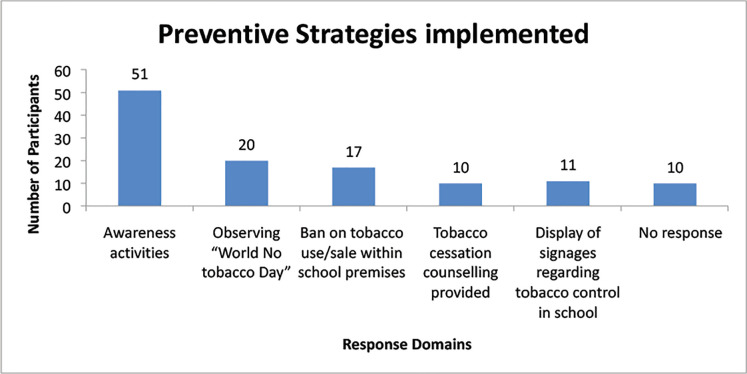
What tobacco preventive strategies are being implemented in your school?

Students were asked regarding the current tobacco control strategies presently implemented in their schools (Figure 2); 62.2% (*N* = 51) informed the role of various health promotional activities like role plays, special assemblies, smart classes, competition, etc., being conducted regularly in their schools.

Students were further probed to suggest possible ways through which they can actively contribute towards tobacco control strategies (Table 4). Most (80.5%; *N* = 66) of the participants were interested in organising tobacco awareness camps at the local level.

## Discussion

Tobacco use amongst youth is escalating in grave proportions across the world. It is believed to achieve the dimension of a pandemic resulting in enormous morbidity and mortality in both developed and developing nations. Various socio-cultural factors influence tobacco consumption patterns especially amongst youth [15]. It has been reported that the majority of the population commence tobacco use habit well before 18 years of age [16].

Hence, the present study provided a qualitative insight into the perception of female youth. The data obtained would help us analyse the existent lacunae in the implementation of tobacco control strategies.

### Knowledge regarding tobacco use and tobacco control strategies

Our results showed that the majority of the participants were aware of the deleterious effects of tobacco on health and emphasised that tobacco may cause cancer. Similar results were reported by Nazir *et al* [17] and Ng *et al* [18] although the study settings and study populations were different. This highlights the knowledge acquired either in schools, through mass media, or through peers which indicates positive baseline behaviour towards tobacco control [18]. It was interesting to note that few of the respondents (4.8%) were concerned about the effect of tobacco use on families as well. Responses highlighted that few of the students were well aware of the future implications of tobacco on families, society and the environment as a whole. This finding can prove quite useful in using this special group in spreading the message to other vulnerable groups.

Regarding tobacco control strategies; 58.6% of the respondents were unaware of various tobacco-related legislations which are in effect in our country. In a previous study also done by Bhambal *et al* [19]; males were more aware regarding legislation against tobacco than females. It was observed that participants were not well aware of the various subsections of COTPA. None of the participants was aware of the subsection of COTPA regarding the ban on the sale of tobacco near school premises.

Lack of tailor-made school-based tobacco-related curriculum can be one of the major contributing factors towards it. Such anti-tobacco campaigns can be an effective strategy to curb tobacco use amongst young people [20].

### Perceived barriers with effective tobacco control strategies

The study findings further analysed the perceived roadblocks regarding the implementation and compliance to tobacco control strategies in our country. This becomes further important when dealing with youth as they are the foundation stone of the health policy implementation of a nation. Females might have a different opinion than males due to various socio-cultural factors.

As per the findings of the Global Adult Tobacco survey; 92.4% of Indian adults believed smoking results in serious illness; still 28.6% of them consume tobacco. Such a significant number of tobacco consumers can be indirectly related to the ineffective implementation of tobacco laws in our country [6]. Responses like ***The laws will not be effective until there are strict punishments for the violation*** and ***people take laws casually until there are strict implications on the violation*** further emphasise the futility of the tobacco control strategies and measures. Around 6% of the participants mentioned the effect of socio-cultural factors as well. Egbe *et al* [21] also found that social-cultural practices influence tobacco usage and exposure. It is, therefore, advocated that tobacco control policies should include culturally themed interventions like street plays, folklores, etc., for spreading awareness.

### Contribution towards tobacco control strategies at the school level

Most of the study participants advocated that various tobacco control strategies and programmes should be dissipated at the school level. The Centre for Disease Control has also led down guidelines for school health programmes to prevent tobacco use and addiction. They have suggested that school programmes designed to prevent tobacco use could become one of the most effective strategies available to reduce tobacco use amongst youth as they bring about positive behaviour change at an early age with life-long impact [22].

It was encouraging to know that the study participants wanted to contribute actively towards tobacco control through street plays, self-control, preparing Information, Education and Communication material, etc. In an interesting response; it was amazing to note that*** I would work towards a complete ban on tobacco products*** showed that the youth is thinking towards a tobacco-free world.

The present study gave a thoughtful insight into the values concerning tobacco use and tobacco control strategies amongst female youth. An attempt has been made to understand the perceived challenges as well. The willingness of the study participants towards active participation in tobacco control strategies is a positive outcome, which can guide in formulating target-specific tobacco control measures. Although the outcome cannot be generalised as it reflects the personal choices and decisions of every respondent; which is the major limitation of the study, the study still provided some considerate information on the students’ engagement towards tobacco control. Future research on the effectiveness of school-based tobacco control programmes would be useful. More of these studies with larger samples and in culturally different population regions should be conducted to provide robust information for future policy decisions.

## Conclusion

The findings from this study substantiated the focus on school-based tobacco control strategies. The results highlighted that our youth is well appraised regarding the impending danger of tobacco use on the health of individuals as well as society as a whole. Dynamic involvement of this vulnerable group in tobacco control policies can help to ensure a tobacco-free future for our coming generations. Strict vigilance and law enforcement are the need of the hour.

## Conflicts of interest

The author(s) declare that they have no conflicts of interest.

## Source of funding

This research received no specific grant from any funding agency in the public, commercial or not-for-profit sector.

## Figures and Tables

**Table 1A.** ‘Tobacco and health’. Please explain.

Response domain	*N*	%
Tobacco is dangerous to health	68	82.9
Tobacco causes cancer	38	46.3
Causes lung diseases	6	7.3
Causes heart diseases	3	3.6

**Table 1B.** ‘Tobacco and health’. Please explain.

Response domain	*N*	%
Tobacco is dangerous to health	68	82.9
Tobacco causes cancer	38	46.3
Causes lung diseases	6	7.3
Causes heart diseases	3	3.6

**Table 2.** Answer in detail why do you think the tobacco control strategies are not effective in our country?

S.no.	Response domains	*N*	%
	No strict tobacco control laws/punishment existent	26	31.7
	Ignorance/lack of awareness amongst people	20	24.3
	Illiteracy	4	4.8
	Increase in tobacco production in India	10	12.1
	Increase in tobacco users	7	8.5
	Decrease in tobacco users	7	8.5
	Complete ban on tobacco is required	2	2.4
	Corruption in implementing agencies	8	9.7
	Culturally imbibed habit	5	6
	No comments	18	21.9

**Table 3.** Why do you think that information regarding tobacco and its effect on health should be dissipated at the school level?

S.no.	Response domains	*N*	%
	To create awareness and bring positive behaviour change	36	43.9
	Students are the nation’s future	14	17
	Teenagers/students start consuming tobacco at an early age	21	25.6
	No response	19	23.17

**Table 4.** As a student how can you contribute towards tobacco control in our country?

S. no.	Response domains	N	%
	Organise awareness campaigns (role-plays/debates/surveys, etc.)	66	80.5
	Prepare and distribute posters/pamphlets, etc.	16	19.5
	Provide tobacco cessation counselling	5	6
	Abstaining from tobacco products	6	7.3
	Advocate complete ban on tobacco	11	13.4
	No reply	1	1.2
	No comments	18	21.9
